# Paeoniflorin Regulates NEDD4L/STAT3 Pathway to Induce Ferroptosis in Human Glioma Cells

**DOI:** 10.1155/2022/6093216

**Published:** 2022-12-28

**Authors:** Xiao-Hu Nie, Sheng Qiu, Ying Xing, Jie Xu, Bin Lu, Shu-Fa Zhao, Yun-Tao Li, Zhong-Zhou Su

**Affiliations:** ^1^Department of Neurosurgery, Huzhou Cent Hospital, Affiliated Cent Hospital HuZhou University, Huzhou 313000, Zhejiang, China; ^2^Department of Gastroenterology, 72nd Group Army Hospital, Huzhou University, Huzhou 313000, Zhejiang, China; ^3^Department of Neurosurgery, Renmin Hospital of Wuhan University, Wuhan 430060, Hubei, China

## Abstract

**Background:**

Paeoniflorin is an active component of a widely used traditional Chinese medicine with antitumor activity through ferroptosis induction. It has been reported recently that ferroptosis is emerging in certain types of cancer; however, its relevance in glioma is still not well studied.

**Methods:**

CCK8 assay was performed for cell proliferation. Expression of mRNA and protein was tested by qPCR and western blot, respectively. Clinical section samples were detected by IHC. The relationship between NEDD4L and STAT3 was validated by a coimmunoprecipitation assay. Apoptosis was identified by TUNEL assay. A xenograft mouse model was utilized to validate the potential of paeoniflorin toward glioma cancer cells.

**Results:**

The data suggested that paeoniflorin could increase NEDD4L expression in glioma cells. The NEDD4L expression level was lower in glioma cancer tissues compared to adjacent normal tissues, and it correlates with poor prognosis. Meanwhile, NEDD4L mediates the ubiquitination of STAT3. Furthermore, increased NEDD4L significantly inhibited cell viability and induced accumulation of intracellular ROS levels, accompanied by decreased expression of key ferroptosis factors Nrl2 and GPX4, while NEDD4L knockdown had a reverse effect, suggesting that ferroptosis could be involved. NEDD4L-induced ferroptosis could be rescued by forced expression of STAT3. A xenograft nude mouse model showed that paeoniflorin inhibits tumor growth and further sensitizes glioma cells to RSL3, another well-known ferroptosis inducer.

**Conclusions:**

In summary, this study demonstrated that paeoniflorin might function as an effective drug for glioma by inducing ferroptosis via upregulation of NEDD4L and repression of Nrl2, GPX4, and STAT3.

## 1. Introduction

Glioma is the most common primary brain tumor derived from the human central nervous system [1]. Although great efforts have been made to improve the prognosis of glioma in the past 40 years, limited advancement has been achieved, especially in patients with high malignancy [2]. New modalities of cancer therapy targeting glioma has always been essentially needed. Our recent discovery demonstrated that paeoniflorin (PF) shows promising antitumor potential in glioma cells [3]. PF is the active ingredient of *Paeonia lactiflora* Pall. (aka P. alba), a traditional Chinese medicine. Recent studies have shown that PF may be an effective antitumor drug for various types of cancers. For example, PF induces cell cycle arrest in HT29 colorectal cancer through p53/14-3-3 zeta pathway [4]. In addition, studies have found that PF can enhance 5-fluorouracil-induced apoptosis in human gastric cancer cells by inhibiting the NF-*κ*B pathway [5].

So far, the mechanism of how PF functions in glioma has not been reported. However, our previous study suggested that the ubiquitination level of STAT3 was elevated in PF-treated glioma cells [3]. The signal transducer and activator of transcription 3 (STAT3) is well studied as a transcription factor that couples extracellular stimulus signals and gene expression. It is known that STAT3 is involved in multiple vital cellular processes such as cell cycle, angiogenesis, and apoptosis [6, 7]. As an important cancer protein during tumorigenesis, the function of STAT3 has been studied extensively [8–10]. It has been reported that the expression as well as activation of STAT3 are also associated with the low survival rate and poor prognostic outcome of glioma patients [11, 12]. Therefore, STAT3 is apparently an attractive target for the treatment of glioma. It has been demonstrated that inhibition of STAT3 through various approaches can trigger growth arrest as well as apoptosis of glioma cells [13–15]. In addition, it has been reported that STAT3 enhances the antioxidant capacity of cells by activating the nuclear factor erythroid 2-related factor 2 (Nrf2). Nrf2 is a transcription factor that prevents oxidative damage by regulating the expression of multiple antioxidant proteins [16]. It has been reported that reactive oxygen species (ROS) can cause aberrant oxidation of molecules such as proteins, lipids, and DNA. Therefore, cancer cells evolved to develop an antioxidant defense system to handle elevated ROS levels in order to successfully establish tumors, which makes the efficient evasion of cell death one of the most important hallmarks of cancer [17–19]. As a nonapoptotic form of regulated cell death relative to apoptosis, ferroptosic cells are typically necrosis-like morphologically with the iconic accumulation of lethal levels of iron-dependent lipid ROS biochemically. Multiple oxidative and antioxidant mechanisms control the oxidative damage during ferroptosis. It has been reported that STAT3/Nrf2 signal pathway may be involved in the regulation of ferroptosis [20]. Cells undergoing ferroptosis are different from apoptosis, necrosis, and autophagy in terms of cell morphology, protein expression, as well as gene expression levels. Ferroptosis has gradually become a hot spot in scientific research after the concept was first proposed in 2012. According to the publications, Erastin and RSL3 are inducers of ferroptosis [21, 22]. Glutathione peroxidase 4 (GPX4) is the key regulatory molecule for ferroptosis, and the reduced expression or activity of GPX4 can induce ferroptosis [22]. Therefore, we proposed to investigate whether ferroptosis is involved in PF-triggered cell death in glioma by examining the aforementioned factors.

On the other hand, given that PF can promote the ubiquitination of STAT3 according to our previous study [3], we sought to screen and identify the ubiquitinase (SYVN1, NEDD4L, CBL, and SOCS5) that bind and mediate ubiquitination of STAT3 based on our data analysis result. We found that PF significantly promotes the expression of human NEDD4L. As a member of an E3-ubiquitin protein ligase NEDD4 family, NEDD4L is expressed in various types of cancer cells and may have carcinogenic properties [23, 24]. By analyzing the cells, array, and RNA sequencing data of TCGA glioma patients, we found that NEDD4L is expressed at a relatively low level in gliomas [25]. In addition, it has been reported that the downregulation of NEDD4L is associated with the progression and malignance of glioma [23]. Furthermore, overexpression of NEDD4L induces glioma cell death [25]. However, the mechanism of how NEDD4L drives these phenotypes are well known. Therefore, this project aims to study the effect and mechanism of PF/NEDD4L/STAT3 on the proliferation of glioma.

## 2. Method and Materials

### 2.1. Cell Culture

Glioma cell lines U251 and U87 were maintained in DMEM medium (Hyclone, SH30243.01; Logan, UT, US) with 10% FBS (GIBCO, 16000e044; Carlsbad, CA, USA) supplement, an extra 1% penicillin-streptomycin (Solarbio, P1400, Beijing, China) was added, and cells were cultured at 37°C in 5% CO_2_ incubator.

### 2.2. Plasmid Construction

The coding sequence of NEDD4L (NM_001144966.2) and STAT3 (NM_139276.3) were synthesized by using the primers containing the restriction enzyme cutting sites of EcoR I and BamH I, and inserted into the pLVX-Puro vector to generate NEDD4L and STAT3 overexpression plasmid which are as follows:

  NEDD4L-F: 5′-CGGAATTCATGGAGCGACCCTATACATTTAAG-3′ (EcoR I)
  NEDD4L-R: 5′-CGGGATCCTTAATCCACCCCTTCAAATCC-3′ (BamH I)
  STAT3-F: 5′-CGGAATTCATGGCCCAATGGAATCAGC-3′ (EcoR I)
  STAT3-R: 5′-CGGGATCCTCACATGGGGGAGGTAGCG-3′ (BamH I)

NEDD4L interference sequences (shown in Table 1) were cloned into the pLKO.1-puro plasmid to downregulate NEDD4L expression.

### 2.3. Cell Transfection

When in the logarithmic growth phase, U251 or U87 cells were trypsinized and seeded in a 6-well plate at 2 × 10^6^ cells/well. Cells were incubated at 37°C overnight in a 5% CO_2_ incubator. When the cells grow to 60–70% confluency, U251 or U87 cells were transfected with oeNEDD4L (MOI = 5, 5 *μ*l) and empty plasmids (vector, MOI = 5, 5 *μ*l), or shNEDD4L-1, shNEDD4L-2, shNEDD4L-3 (MOI = 5, 5 *μ*l), and shNC (MOI = 5, 5 *μ*l), or oeSTAT3 (MOI = 5, 5 *μ*l), and empty plasmids (vector, MOI = 5, 5 *μ*l) using transfection reagent Lipo2000 based on the manufacturer's protocol. 24 hours after transfection, the serum-free medium was replaced by a complete medium, cells were then incubated for 48 hours.

### 2.4. Cell Counting Kit-8 (CCK-8) Assay

Cell proliferation was accessed by CCK-8 assay using a Cell Proliferation and Cytotoxicity Assay Kit (SAB, CP002; College Park, MD, USA) in accordance with the manufacturer's protocol. Briefly, 100 ul of a solution containing 2 × 10^3^ U251 or U87 cells was seeded in each well of a 96-well plate and incubated overnight. The cells were then distributed into various treatments. Afterwards, 10 *μ*l of CCK-8 solution was then added to each well. Absorbance at 450 nm of each well was measured in a plate reader to evaluate cell viability.

### 2.5. Biochemical Detection

The levels of malonyl dialdehyde (MDA) and Fe^2+^ in cells were, respectively, measured using the MDA (A003-4-1, Nanjing, Jiancheng Biotechnology Research Institute, Jiangsu, China) and Iron assay (ab83366, Abcam) Kit according to the manufacturers' instructions. Assays were performed in triplicate, and the mean values of each sample were calculated manually.

### 2.6. Quantitative Real-Time PCR (qRT-PCR)

The mRNA levels were determined by qRT-PCR. Total RNA was extracted with TRI reagent (Sigma T9424). cDNA was generated using the RevertAid First Strand cDNA Synthesis Kit (Fermentas, Hanover, MD, USA). SYBR Green qPCR Master Mixes (#K0223, Thermo Fisher, Rockford, IL, USA) was utilized for qPCR according to the manufacturers' instructions. The relative mRNA levels were normalized to GAPDH and calculated as 2^−ΔΔCt^ . The primers used are listed in Table 2.

### 2.7. Immunoprecipitation (IP) Detection

The total proteins were grouped to incubate with 1 *μ*g of Rabbit-IgG (Sc-2027, Santa Cruz Biotechnology) and 1 *μ*g of IP-indicated antibody at 4°C overnight, while untreated proteins were used as an input control, followed by incubation of Protein A/G PLUS-Agarose for 2 hours at room temperature to form an immune complex. After centrifuging for 4 min at 3000 rpm at 4°C, 1 ml of lysis buffer was added to wash the Protein A/G Plus-Agarose beads 3 times, and appropriate protein loading buffer was added and boiled for 5 min to elute the immunoprecipitates. Following centrifugation 3000 rpm for 1 min, the supernatant was collected for western blot analysis. The antibodies of anti-NEDD4L (Ab46521, Abcam) and anti-STAT3 (Ab32500, Abcam) were applied for IP detection, while anti-NEDD4L (13690-1-AP, Proteintech) and anti-STAT3 (10253-2-AP, Proteintech) were used in western blot analysis.

### 2.8. Western Blot Analysis

To determine protein level, the sample cell lysates were separated with SDS polyacrylamide gels. Proteins of interest were transferred to polyvinylidene fluoride membranes which were then blocked with 5% nonfat milk for at least one hour. The membranes were then blotted with optimized primary antibodies overnight at 4 C and secondary antibodies for one hour at room temperature, respectively. Protein abundance was assessed using a chemiluminescent imaging system (Tanon 5200, Shanghai, China). Anti-SYVN1 (Ab170901), anti-CBL (Ab32027), anti-SOCS5 (Ab97283), anti-NEDD4L (Ab46521), anti-GPX4 (Ab125066), anti-STAT3 (Ab68153), anti-p-STAT3 (Ab76315) supplied from Abcam, and anti-Nrf2 (16396-1-AP) and anti-GAPDH (60004-1-1G) obtained from Proteintech were used.

### 2.9. Measurement of Intracellular ROS Levels

The intracellular ROS levels were measured using a Reactive Oxygen Species Assay Kit (S0033, Beyotime). 2′,7′-dichlorofluorescein-diacetate (DCFH-DA), which is easily oxidized to fluorescent dichlorofluorescein (DCF) by intracellular ROS, is its principal component, and therefore, the ROS levels were quantified. Briefly, the cell pellets were collected after receiving various treatments and then resuspended in 1 ml of cool PBS. A 10 *μ*M probe staining working solution was prepared by diluting 10 mM DCFH-DA probe solution to 1 : 1000 in serum-free medium. Cells were then incubated with DCFH-DA probe solution in the dark at 37°C for 30 min. The tube was inverted every 5 min to mix cells and solution. Samples were washed with serum-free medium thrice and then measured at 488 nm excitation and 525 nm emission by a flow cytometer (CytoFLEX, Beckman).

### 2.10. Immunohistochemistry (IHC)

Tissue slices were first rinsed with 0.02 M PBS three times and fixed in 4% paraformaldehyde solution (10010018, National Medicines Corporation Ltd.) for 30 min. Next, the slides were permeabilized with 3% H_2_O_2_ (10011218, Shanghai Sinopharm) for 10 min, then were blocked in 1% bovine serum albumin (A8010, Solarbio) for one hour at room temperature. Afterwards, the tissue slices were incubated with primary antibodies against NEDD4L (13690-1-AP, Proteintech) and STAT3 (Abcam, ab68153) in a humidifying box for 1 hour at room temperature. The tissue slices were rinsed with PBST thrice to remove any residual antibodies. Next, the tissue slides were incubated in HRP-labeled secondary antibodies (D-3004, Changdao) for 30 min at room temperature. The slides were stained with DAB (FL-6001, Changdao), and then rinsed with tap water to remove the staining solution. The slides were counterstained with hematoxylin (714094, BASO) for 3 min, followed by incubation with 1% hydrochloric acid for alcohol differentiation. The slides were then washed with tap water for 10 min and then subsequently subjected to a grill, transparent and closed, after which the slices were imaged under a microscope (ECLIPSE Ni, NIKON). The IHC results were accessed by two experienced pathologists independently. The staining signal was graded as 0, negative; 1, weakly positive; 2, moderate positive; and 3, strong positive. The percentage of positive cells was scored as 0, < 5%; 1, 5%–25%; 2, 25%–50%; 3, 50%–75%; and 4, > 75%. The staining index was defined as follows: staining index = staining intensity × percentage of positive staining cells. The sample was categorized as a high expression if the staining index was higher than 3.

### 2.11. *In Vivo* Experiments

U251 cells (6 × 10^6^) were inoculated into the flanks of 4-to 5-week-old athymic nude mice (Shanghai Laboratory Animal Company, Shanghai, China) subcutaneously to generate a subcutaneous xenograft tumor model. After 2 weeks, the tumor model was successfully constructed, the mice were treated single and combined with 100 mg/kg RSL3 (2 times/week) and 1.0 g/kg/days PF. Tumor volumes were measured every 4 days to draw the growth curve. Mice were sacrificed 4 weeks after cell injection. Tumor xenografts were collected, photographed, and weighed and the tumor apoptosis was analyzed by Tunel staining. All care and experiment of the laboratory animals were performed following protocols approved by Huzhou Cent Hospital, Affiliated Cent Hospital Huzhou University (Zhejiang, China).

### 2.12. Detection of Apoptosis

To detect apoptotic cells, the terminal deoxynucleotidyl transferase-mediated deoxyuridine triphosphate biotin Nick end labelling (TUNEL) method was conducted. In brief, specimens were first dewaxed and rehydrated, then incubated in proteinase K (40 *μ*g/ml) at 37°C for one hour. The slides were then treated with 3% H_2_O_2_ (diluted in methanol) for 30 minutes at room temperature. After washing with PBS, sections were incubated with equilibration buffer for five minutes at room temperature, then incubated with the terminal deoxynucleotidyl transferase enzyme at 37°C for two hours. The sections were then incubated with 100 ul of stop buffer for 30 minutes at 37°C. The slides were covered in anti-digoxigenin peroxidase for 30 minutes at 37°C. Slides were then visualized with diaminobenzene and counterstained with hematoxylin. 500 cells in total were counted for each section. The apoptotic index was calculated as a ratio of apoptotic cell number/total cell number times 100. Tumor specimens were categorized according to TUNEL staining into ≤10% or >10% stained cells.

### 2.13. Statistical Analysis

All statistical analyses in the current project were conducted in GraphPad Prism 7.0 software (San Diego, CA, USA). At least three independent repetitive experiments were performed for each result. The data were presented as a mean value ± SD. One-way analysis of variance (ANOVA) with Tukey's posthoc tests were applied for the comparison of mean values. *p* < 0.05 was regarded as statistically significant.

## 3. Result

### 3.1. Treatment of PF Significantly Increases NEDD4L Expression in Glioma Cells

**Figure 1:**
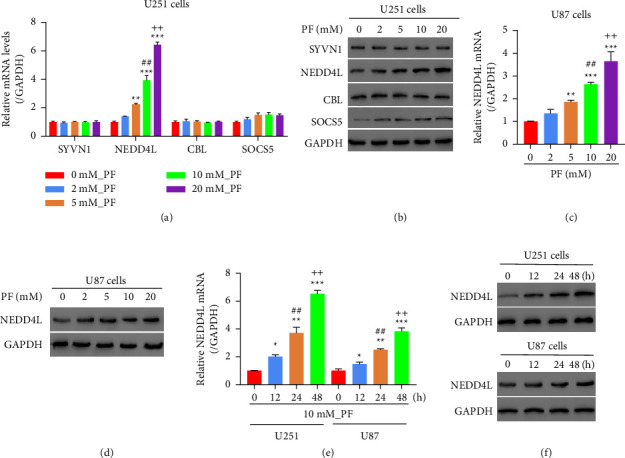
Treatment of paeoniflorin significantly increased NEDD4L expression in glioma cell lines U251 and U87. (a)-(b) U251 cells were treated with PF at indicated concentration for 24 hours, after which mRNA and protein levels of SYVN1, NEDD4L, CBL, and SOCS5 were determined by qPCR (a) and western blot (b). (c)-(d) U87 cells were treated with PF at indicated concentration for 24 hours, after which mRNA and protein levels of SYVN1, NEDD4L, CBL, and SOCS5 were determined by qPCR (c) and western blot (d). (e)-(f) U251 cells and U87 cells were treated with 10 mM PF for different times as indicated, after which mRNA and protein levels of NEDD4L were determined by qPCR (e) and western blot (f).

Our previous study demonstrated that PF can promote the ubiquitination of STAT3 [3]. To identify the potential ubiquitinase that carries out the STAT3 ubiquitination, at first, we selected 4 candidates (SYVN1, NEDD4L, CBL, and SOCS5) that can bind with STAT3 through data analysis. And then the expression of these 4 candidates in U251 cells in response to PF treatment were examined at both mRNA and protein levels. We noticed that PF only promotes the expression of NEDD4L significantly in a dose-dependent manner without affecting the other 3 ubiquitinases (Figures 1(a) and 1(b)). To verify this observation, we examined the expression level of NEDD4L in another glioma cell line U87 upon the treatment of PF. In U87 cells, the expression of NEDD4L is increased gradually as the dosage of PF increases (Figures 1(c) and 1(d)). In addition, PF promotes the expression of NEDD4L in a time-dependent manner in glioma cells as well (Figures 1(e) and 1(f)). Taken together, our data suggest that PF may induce STAT3 ubiquitination by promoting the expression of NEDD4L.

### 3.2. NEDD4L is Lowly Expressed in Glioma and Associated with the Prognosis of Patients

**Figure 2:**
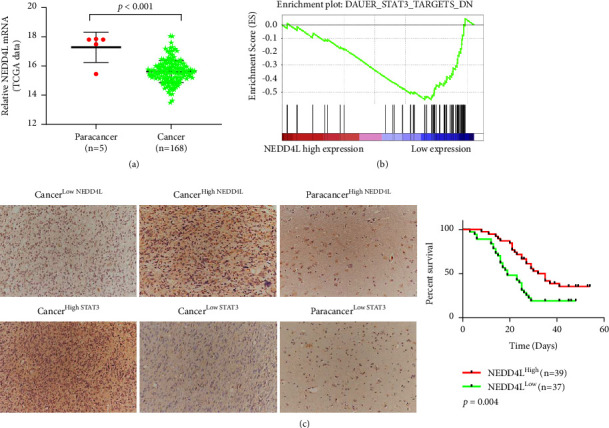
NEDD4L was lowly expressed in glioma and is associated with the prognosis of patients. (a) Expression of NEDD4L in TCGA glioma and normal tissue samples. (b) GSEA analysis of NEDD4L high and low TCGA samples showed negative correlation between NEDD4L expression and STAT3 signaling pathway. (c) IHC staining of NEDD4L and STAT3 in glioma tissue chip (left) and Kaplan-meier survival analysis of NEDD4L high and low glioma patients (right).

In order to explore the potential roles of NEDD4L in glioma, we first compared the expression of NEDD4L in 168 cancer samples and 5 normal tissue from the TCGA database. The data showed that NEDD4L is significantly downregulated in gliomas (Figure 2(a)). In addition, we carried out a gene set enrichment analysis (GSEA) to identify the signaling pathways that are significantly altered along with the level of NEDD4L. It turned out that NEDD4L negatively regulates the activation of the STAT3 pathway (Figure 2(b)). In order to verify the expression levels of NEDD4L and STAT3 in glioma tissue, we performed immunohistochemical staining of NEDD4L and STAT3 on a commercially available glioma tissue chip, and found that NEDD4L expression level negatively correlates with STAT3 expression in glioma tissues, while the expression levels of these two genes are comparable in normal tissue (Figure 2(c) left panel). In addition, Kaplan-Meier analysis showed that patients with NEDD4L-low-expressing glioma exhibit a worse prognosis compared with patients with NEDD4L-high-expressing glioma (Figure 2(c) right panel), which demonstrates the potential of NEDD4L function as a biomarker to predict the clinical outcome of glioma patients.

### 3.3. NEDD4L Binds to STAT3 and Induces its Ubiquitination in Glioma Cells

**Figure 3:**
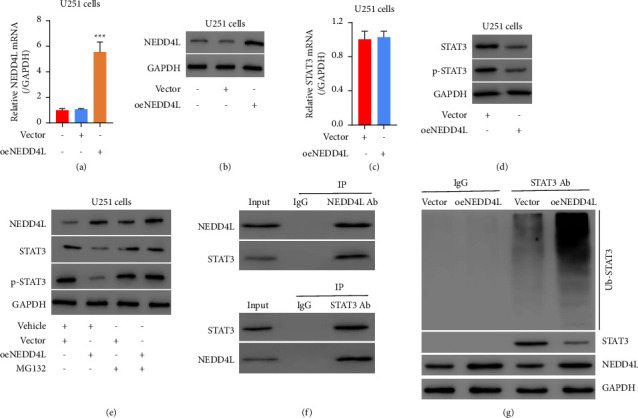
The interaction between NEDD4L and STAT3 in glioma cell line. (a)-(b) U251 was transfected with either oeNEDD4L or vector plasmid and the efficiency was validated by qPCR (a) and western blot (b). ^*∗∗∗*^*p* < 0.001 vs. vector. (c) mRNA level of STAT3 in U251 cell overexpressing NEDD4L was tested by qPCR. (d) Western blot of total and phosphor-STAT3 in U251 cells overexpressing NEDD4L. (e) Western blot of NEDD4L, STAT3, and phosphor-STAT3 in cells overexpressing NEDD4L with or without MG132 treatment. (f) Coimmunoprecipitation of NEDD4L and STAT3. (g) Ubiquitination of STAT3 is dramatically increased in NEDD4L overexpressing U251 cells. Western blot of STAT3 and NEDD4L was set for reference.

In order to determine the interaction between NEDD4L and STAT3 in glioma cells, we engineered glioma U251 cells to overexpress NEDD4L, which was validated by both qPCR and western blot (Figures 3(a) and 3(b)). Then, it turned out that forced expression of NEDD4L significantly inhibits the protein levels of the total as well as phosphorylated STAT3 (Ser727) without affecting the level of STAT3 mRNA (Figures 3(c) and 3(d)). On the other hand, this process is dramatically inhibited by the treatment of MG132, suggesting that NEDD4L could downregulate the expression level of STAT3 through the protein degradation pathway (Figure 3(e)). We then carried out a coimmunoprecipitation (Co-IP) assay to determine whether STAT3 interacts with NEDD4L in glioma U251 cells. As we expected, the binding between NEDD4L and STAT3 was elucidated by both forward and reverse IP (Figure 3(f)). Furthermore, forced expression of NEDD4L significantly increases the ubiquitination of STAT3 (Figure 3(g)).

### 3.4. Forced Expression of NEDD4L Significantly Inhibits Proliferation and Increases Intracellular ROS Levels in Glioma Cells, Whereas NEDD4L Downregulation has a Reverse Effect

**Figure 4:**
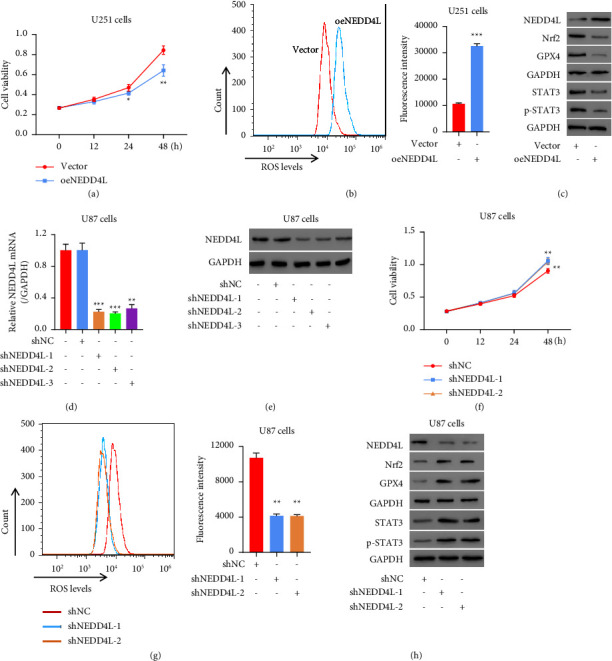
Up-regulation of NEDD4L significantly inhibited proliferation and increased intracellular ROS levels in glioma cell line, whereas NEDD4L down-regulation had a reverse effect. (a) Cell proliferation of U251 overexpressing NEDD4L was tested by CCK8 assay. (b) Intracellular ROS levels of U251 oeNEDD4L was tested by flow cytometry. (c) Western blot of NEDD4L, STAT3, phosphor-STAT3, Nrf2, and GPX4. ^*∗*^*p* < 0.05, ^*∗∗*^*p* < 0.01, ^*∗∗∗*^*p* < 0.001 vs. vector. (d)-(e) NEDD4L in U87 cells was knockdown by three shRNAs, and the knockdown efficiency was validated by qPCR (d) and western blot (e). (f) U87 cell proliferation was tested by CCK8 after knocking down NEDD4L with two shRNA respectively. (g) Effect of knocking down NEDD4L on intracellular ROS levels in U87 cells was examined by flow cytometry. ^*∗∗*^*p* < 0.01 vs. shNC. (h) Protein level of Nrf2, GPX4, STAT3, phosphor-STAT3, and NEDD4L were tested by western blot.

As suggested by our previous data that a high level of NEDD4L improves the overall survival of glioma patients, we proposed to investigate whether the manipulation of NEDD4L affects glioma cell proliferation. We generated a U251 NEDD4L-overexpression (oeNEDD4L) cell line as mentioned above and performed a CCK8 assay to examine the cell proliferation once every 12 hours for 48 hours. It turns out that forced expression of NEDD4L prohibits cell proliferation significantly 24 hours after transfection (Figure 4(a)). In addition, the intracellular ROS levels were increased (Figure 4(b)). Meanwhile, the expression of Nrf2 and GPX4, two well-known key factors of a ferroptosis signaling pathway, was suppressed (Figure 4(c)). Meanwhile, the levels of total and phosphorylated STAT3 are dramatically decreased. In order to further validate this observation, we generated NEDD4L-knockdown (shNEDD4L) U87 glioma cell lines using three independent shRNAs targeting NEDD4L. As shown in Figures 4(d) and 4(e), the expression of NEDD4L is significantly prohibited at both mRNA and protein levels. Contrary to the oeNEDD4L cells, proliferation is promoted in the NEDD4L-knockdown glioma cells (Figure 4(f)). Likewise, the intracellular ROS levels were significantly decreased after knocking down NEDD4L (Figure 4(g)). The expressions of Nrf2, GPX4, STAT3, as well as phosphorylated STAT3 all increased, while the NEDD4L expression decreased after shRNA knocking down (Figure 4(h)). These data suggest that NEDD4L negatively regulates key ferroptosis factors as well as STAT3.

### 3.5. Upregulation of STAT3 Significantly Reversed NEDD4L-Induced Proliferative Inhibition and Intracellular ROS Levels Elevation in Glioma Cells

**Figure 5:**
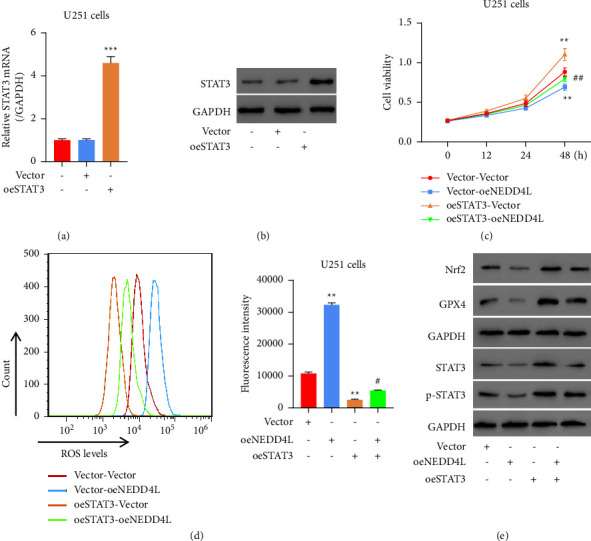
Upregulation of STAT3 significantly reversed NEDD4L-induced proliferation inhibition and intracellular ROS levels in glioma cell line. (a)-(b) U251 was transfected with oeSTAT3 plasmid to overexpress STAT3, and the expression of STAT3 was tested by qPCR (a) and western blot (b). ^*∗∗∗*^*p* < 0.001 vs. vector. (c) Cell proliferation of U251 overexpressing either or both NEDD4L and STAT3 was tested by CCK8 assay. (d) Intracellular ROS levels in U251 overexpressing either or both NEDD4L and STAT3 were tested by flow cytometry. ^*∗∗*^*p* < 0.01 vs. vector_vector; ^#^*p* < 0.05, ^##^*p* < 0.01 vs. vector_oeNEDD4L. (e) Western blot of Nrf2, GPX4, STAT3, and phosphor-STAT3 in U251 overexpressing either or both NEDD4L and STAT3.

In order to investigate whether NEDD4L regulates glioma cell proliferation through manipulating STAT3, we generated STAT3-overexpressing U251 glioma cells, and the overexpression of STAT3 was validated by both qPCR and western blot (Figures 5(a) and 5(b)). Furthermore, we induced overexpression of STAT3, either alone (oeSTAT3) or together with NEDD4L overexpression (oeSTAT3-oeNEDD4L). Interestingly, we noticed that the suppression of cell proliferation induced by oeNEDD4L is rescued by cooverexpression of STAT3 (Figure 5(c)). Consistent with this observation, the elevated intracellular ROS levels induced by NEDD4L overexpression were also rescued by forced expression of STAT3 (Figure 5(d)). Likewise, NEDD4L-induced decreased Nrf2 and GPX4 expression were restored to the normal or even higher levels after overexpression STAT3 (Figure 5(e)). The protein levels of STAT3 and phosphorylated STAT3 are all increased upon the overexpression of STAT3 even in the oeNEDD4L background (Figure 5(e)). Taken together, these data imply that NEDD4L carries out its effect on glioma by manipulating STAT3, which appears to be the downstream factor for the signaling pathway.

### 3.6. Treatment of PF Inhibits Cell Proliferation and Increases Intracellular ROS Levels in Glioma Cells by Regulating NEDD4L Expression

**Figure 6:**
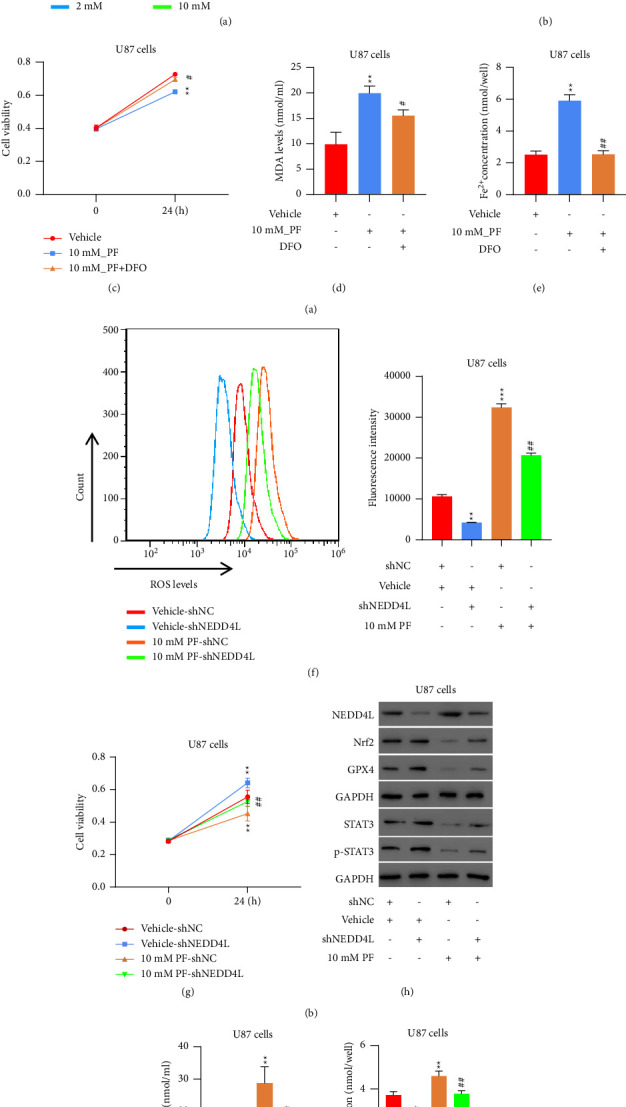
Treatment of paeoniflorin may inhibit proliferation and increased intracellular ROS levels in glioma cell line by regulating NEDD4L expression. (a) Intracellular ROS levels in U87 cells treated with PF as indicated concentration was determined by flow cytometry. ^*∗*^*p* < 0.05, ^*∗∗*^*p* < 0.01, ^*∗∗∗*^*p* < 0.001 vs. 0 mM_PF; ^#^*p* < 0.05 vs. 2 mM_PF; ^++^*p* < 0.01 vs. 5 mM_PF. (b) Expression levels of NEDD4L, Nrf2, GPX2, STAT3, and phosphor-STAT3 in U87 cells treated with PF. (c) Cell proliferation of U87 treated with either or both PF and DFO was tested by CCK8 assay. (d)-(e) Levels of MDA (d) and Fe^2+^ (e) in U87 cells treated with either or both PF and DFO were measured using MDA and iron assay kit. ^*∗∗*^*p* < 0.01 vs. vehicle; ^#^*p* < 0.05, ^##^*p* < 0.01 vs. 10 mM_PF. (f) Intracellular ROS levels of U87 treated with either or both PF and shNEDD4L was tested by flow cytometry. (g) Cell proliferation of U87 treated with either or both PF and shNEDD4L was tested by CCK8 assay. ^*∗∗*^*p* < 0.01 vs. Vehicle_shNC; ^##^*p* < 0.01 vs. vehicle_shNEDD4L. (h) Western blot of NEDD4L, Nrf2, GPX4, STAT3, and phosphor-STAT3. (i)-(j) Levels of MDA (i) and Fe^2+^ (j) in U87 cells treated with either or both PF and shNEDD4L were measured using MDA and Iron assay kit. ^*∗*^*p* < 0.05, ^*∗∗*^*p* < 0.01 vs. vehicle_shNC; ^##^*p* < 0.01 vs. vehicle_shNEDD4L.

As mentioned above, our previous study implied the relationship between PF's antitumor effects and STAT3 ubiquitination. In order to further elucidate the mechanism involved in this process, we first checked the intracellular ROS levels in response to the PF treatment. As shown in Figure 6(a), the intracellular ROS levels increase gradually as the PF dosage increases. In order to determine if ferroptosis is affected by PF treatment, we examined the expressions of STAT3/p-STAT3, Nrf2, and GPX4 in PF-treated glioma U87 cells, and found out that all these factors are inhibited at the protein level in a dosage-dependent manner (Figure 6(b)). Moreover, treatment of PF significantly inhibited cell proliferation (Figure 6(c)) and increased the levels of MDA (Figure 6(d)) and Fe^2+^ (Figure 6(e)) in glioma cells, whereas ferroptotic inhibitors DFO could partly reverse the effect of PF, suggesting that PF treatment can induce ferroptosis in glioma cells. To test this hypothesis that PF might function through the elevation of NEDD4L, we knocked down the NEDD4L in U87 glioma cells for 24 hours before subjecting these cells to PF treatment. As we speculated, following ablation of NEDD4L, the promoted intracellular ROS levels in the cells with PF treatment was decreased (Figure 6(f)), consistent with intracellular ROS levels, ablation of NEDD4L attenuated the growth inhibitory effect of PF (Figure 6(g)). Likewise, the expression levels of Nrf2, GPX4, STAT3, and p-STAT3 (Figure 6(h)), as well as the levels of MDA (Figure 6(i)) and Fe^2+^ (Figure 6(j)) were restored close to normal after receiving PF treatment in the absence of NEDD4L. Taken together, our data suggest that PF carries out its antitumor effect by regulating the expression of NEDD4L, which could further manipulate ferroptosis in glioma cells.

### 3.7. PF Inhibits Tumor Growth by Increasing Ferroptosis of Tumor Cells *In Vivo and In Vitro*

**Figure 7:**
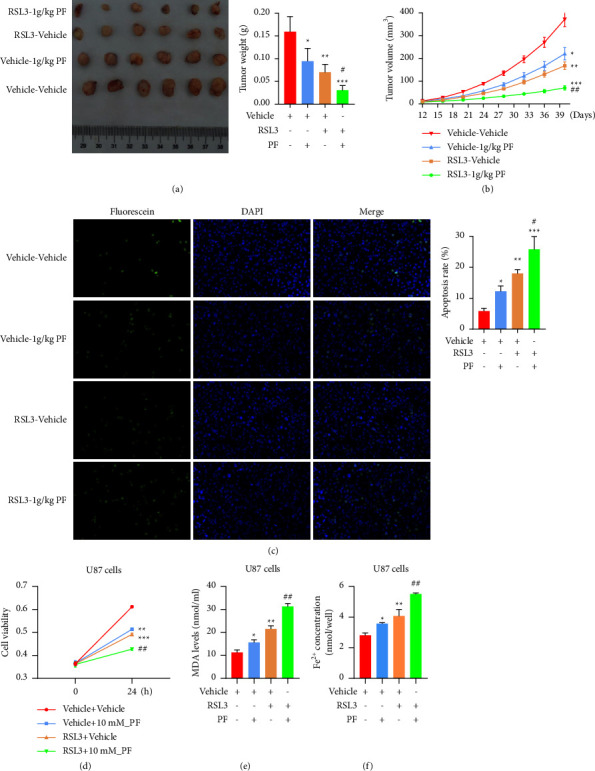
Paeoniflorin may inhibit tumor growth by increasing ferroptosis of tumor cells *in vivo*. U251 xenograft model was established by injecting cells subcutaneously. Mice-bearing tumor 2 weeks after injection was treated with either or both RSL3 (100 mg/kg, twice/week) and PF (1 g/kg/day). (a) Tumor size (left) and weight (right) from each treatment group was exhibited. (b) The growth curves of each treatment group. (c) The apoptosis status of the tumor tissues was determined by TUNEL assay and the quantification of the apoptosis was shown in bar chart. ^*∗*^*p* < 0.05, ^*∗∗*^*p* < 0.01, ^*∗∗∗*^*p* < 0.001 vs. vehicle_vehicle. (d) Cell proliferation of U87 treated with either or both of PF and RSL3 was tested by CCK8 assay. (e)-(f) Levels of MDA (e) and Fe^2+^ (f) in U87 cells treated with either or both of PF and RSL3 were measured using MDA and Iron assay Kit. ^*∗*^*p* < 0.05, ^*∗∗*^*p* < 0.01, ^*∗∗∗*^*p* < 0.001 vs. vehicle_vehicle; ^##^*p* < 0.01 vs. RSL3_vehicle.

Next, we sought to assess the therapeutic effect of PF in the U251 xenograft tumor model. In general, U251 cells were implanted into nude mice subcutaneously, and the mice bearing established tumor 2 weeks after injection and they were subjected to one of the four following treatments: vehicle; RSL3 (100 mg/kg, twice/week), an experimentally verified drug for inducing ferroptosis [26]; PF (1 g/kg/day); concomitant administration of RSL3 and PF. The tumor growth was closely monitored. Our results showed that both monotherapies significantly attenuated tumor growth. Furthermore, the combination of RSL3 and PF results in significantly decreased tumor burden and prolonged progression compared to RSL3 or PF monotherapy (Figures 7(a) and 7(b)). The mice were sacrificed 28 days after receiving treatment. Tumor tissues were collected afterwards and subjected to a TUNEL assay to assess the apoptosis status. Apparently, the PF treatment substantially induces apoptosis which leads to suppressed tumor growth eventually, and enhanced the apoptosis induced by RSL3, which could further sensitize glioma cells to RSL3 treatment (Figure 7(c)). Consistent with the *in vivo* results, the *in vitro* results showed that the combination of RSL3 and PF was superior to RSL3 or PF monotherapy and more significantly inhibited cell proliferation (Figure 7(d)) and increased MDA (Figure 7(e)) and Fe^2+^ (Figure 7(f)) levels in gliomas. Together, our data suggest that the combination of PF and RSL3 may be beneficial to inhibit glioma tumors by inducing ferroptosis.

## 4. Discussion

Finding active components of traditional Chinese medicine and discovering the underlying mechanism have become a hot topic in medicine studies. As an active chemical compound, it is one of the major constituents of Paeonia lactiflora Pall. (a.k.a P. alba), paeoniflorin (PF) exhibits great potential antitumor effects in various types of cancer by targeting different cell events such as cell cycle arrest, apoptosis, migration, and epithelial-mesenchymal transition (EMT) [27–29]. In addition, the anticancer effects of PF have been reported in the context of glioblastoma [30–32]. Our previous study showed that PF inhibits human glioma cell proliferation by inhibiting the STAT3 pathway via regulating its turnover through the ubiquitination pathway [3].

In this study, we pushed forward our knowledge about the therapeutic value of PF and the mechanism that renders PF's antitumor capability. Our data suggest that PF can induce STAT3 ubiquitination by promoting the expression of NEDD4L. As a member of an E3 ligase NEDD4 family, NEDD4L not only targets membrane proteins including ion channels and transporters, but also triggers the degradation of certain proteins involved in cancer signaling pathways such as Dvl2, SMAD2, and SMAD7 [33, 34]. It has been demonstrated that NEDD4L suppresses ferroptosis by mediating the degradation of lactotransferrin (LTF) protein which functions as an activator of ferroptosis [35]. Ferroptosis is defined as a type of oxidative iron-dependent lipid peroxidation-induced programmed cell death. One of the most essential characteristics of ferroptosis is the rapid accumulation of reactive oxygen species (ROS) induced by high levels of cellular labile iron [36]. And the role of ROS in the context of cancer has been well studied. For instance, it has been well established that elevated levels of ROS render disruptive effects on functions and structures of cells which leads to oxidative stress and subsequently to the development of various pathologies including inflammatory, age-related disorders, and cancer [37, 38]. And it is known that ROS play vital roles in mitogenic signaling cascades by prolonging the activation of growth factors and boosting cellular signaling factors [39, 40].

However, the exact role of NEDD4L in glioma is still not fully elucidated. In this study, we noticed that the expression of NEDD4L is upregulated by PF in a dosage- and time-dependent manner. We then found out that NEDD4L is downregulated in glioma cancer tissue compared to normal tissue, which is consistent with published data [25], and the expression level of NEDD4L correlates with prognosis in cancer patients. We also provide solid evidence to show that NEDD4L negatively regulates STAT3 level by mediating its ubiquitination, which bridged the gap between PF and STAT3 as reported in the previous study. The oncogenic roles of STAT3 have been studied extensively in the past few decades [8–10]. It has been reported that STAT3 could be a promising target for the treatment of glioma [41]. It also has been reported that the expression as well as activation of STAT3 are associated with the low survival rate and poor prognosis of glioma patients [11, 12]. In addition, STAT3 has been suggested to participate in glioma cell progression by shaping the immune microenvironment [42]. On the other hand, it has been suggested that STAT3 plays key roles in ferroptosis which demonstrates a new approach to attenuate drug resistance in osteosarcoma [20]. Taken together, these data endorsed the importance of tackling down STAT3 pathway in the battle of fighting glioma.

In addition, our data demonstrated that forced expression of NEDD4L inhibits glioma cell growth probably by inducing ferroptosis, characterized by intracellular ROS accumulation and suppressed expression of the key factors in ferroptosis such as Nrf2 and GPX4. It has been reported that Nrf2 is required for glioma stem cell self-renewal [43–45]. GPX4 is also involved in the proliferation, migration, and apoptosis of glioma cells [46]. Therefore, the reduced expression of Nrf2 and GPX4 driven by oeNEDD4L might contribute to the suppressed glioma cell growth by multiple means. In addition, NEDD4L drives the ubiquitination of STAT3, which also plays critical roles in ferroptosis as previously discussed. Furthermore, our xenograft data demonstrated that PF can suppress glioma tumor growth by activating NEDD4L/STAT3/Nrf2/GPX4 signal axis which eventually triggers ferroptosis. And the antitumor effect could be enhanced by the concomitant administration of PF and RSL3.

It is known that as a ferroptosis inducer, RSL3 inhibits tumor growth by targeting GPX4. Then, PF could enhance the RSL3 treatment by either thoroughly inhibiting GPX4 or inducing both ferroptosis and apoptosis. Therefore, more studies need to be performed to explore the specific underlying mechanism.

## 5. Conclusions

Taken together, this study demonstrates that PF can function as an antitumor agent for glioma treatment by targeting NEDD4L-dependent STAT3 ubiquitination as well as by regulating the Nrf2/GPX4 signaling axis, which might trigger ferroptosis. Furthermore, PF could benefit glioma patients by enhancing antitumor effect of other ferroptosis inducers such as RSL3, which could pave the road for new therapeutic modalities.

## Figures and Tables

**Table 1:** NEDD4L interference sequences.

Name	Sequences
NEDD4L site 1 (481–499)	GAGCGACCCTATACATTTA
NEDD4L site 2 (635–653)	GGGAAGTTGTTGACTCAAA
NEDD4L site 3 (1094–1112)	GCTCTTTGATTCAAAGAGA

**Table 2:** The primers used.

Name	Primers
SYVN1	5′ AGCTGGAGTCTCCTGTTG 3′
	5′ AGAGGAAGGCTGGAACTG 3′

NEDD4L	5′ TCTGGAAGGCTGTGCTAC 3′
	5′ TCTGGGCAGTTTCTCAGG 3′

CBL	5′ AGTGGAGTTGTGGTAAGG 3′
	5′ AGTTGGAAGGTGGTGTAG 3′

SOCS5	5′ TTCACTCCTCCACTGTAACG 3′
	5′ TCCAACCAGCGAACTCTAAC 3′

STAT3	5′ TGTCTAAAGGTCCCTCATC 3′
	5′ CCATAGTGTGCATCATGTC 3′

GAPDH	5′ AATCCCATCACCATCTTC 3′
	5′ AGGCTGTTGTCATACTTC 3′

## Data Availability

The data supporting the current study are given in the article.

## Supplementary Materials

Supplementary Materials Supplementary Figure 1. The expression of NEDD4L in glioma cell line U251 and U87, and 293T cells. Supplementary Figure 2. Treatment of paeoniflorin and NEDD4L upregulation may inhibit proliferation glioma cell line by increasing ferroptosis. U251 cells were treated with Erastin (10 *μ*M) either alone or combined with either PF or transfected with NEDD4L overexpression plasmid for 24 hours. (a) The proliferation of treated cells was determined by CCK8 assay. (b) The lipid ROS level was examined by flow cytometry. (c)-(d) Levels of MDA (c) and Fe^2+^ (d) in U251 cells were measured using MDA and Iron assay Kit. ^*∗∗*^*p* < 0.01 vs. Vehicle; ^##^*p* < 0.01 vs. 10 *μ*M Erastin-Vector.
