# Supplementary material for: Paeoniflorin Regulates NEDD4L/STAT3 Pathway to Induce Ferroptosis in Human Glioma Cells
Source: J Oncol. 2022 Dec 28;2022:6093216. doi: 10.1155/2022/6093216 (PMC9812627; doi:10.1155/2022/6093216)
Supplement: Supplementary Materials — Supplementary Figure 1. The expression of NEDD4L in glioma cell line U251 and U87, and 293T cells. Supplementary Figure 2. Treatment of paeoniflorin and NEDD4L upregulation may inhibit proliferation glioma cell line by increasing ferroptosis. U251 cells were treated with Erastin (10 μM) either alone or combined with either PF or transfected with NEDD4L overexpression plasmid for 24 hours. (a) The proliferation of treated cells was determined by CCK8 assay. (b) The lipid ROS level was examined by flow cytometry. (c)-(d) Levels of MDA (c) and Fe2+ (d) in U251 cells were measured using MDA and Iron assay Kit. ∗∗p < 0.01 vs. Vehicle; ##p < 0.01 vs. 10 μM Erastin-Vector. [file 6093216.f1.pptx]

## Slide 1
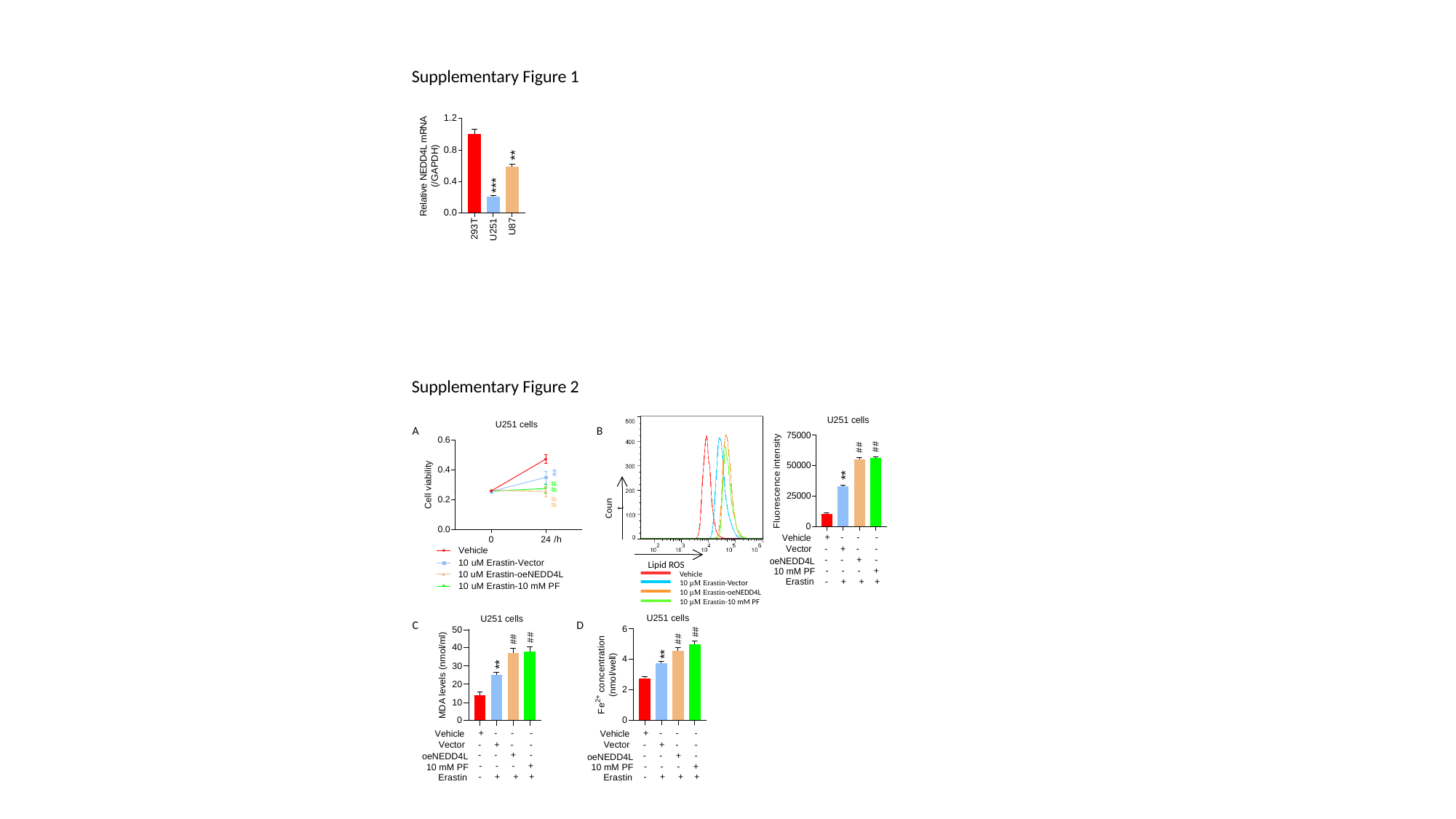

Supplementary Figure 1
Supplementary Figure 2
Count
Lipid ROS
A
B
Vehicle
10 µM Erastin-Vector
10 µM Erastin-oeNEDD4L
10 µM Erastin-10 mM PF
C
D
